# Perioperative normothermia in Asia and Australasia: challenges, implementation strategies, recommendations, and correct use of forced air warming

**DOI:** 10.1186/s13741-026-00680-7

**Published:** 2026-04-15

**Authors:** Justin Sangwook Ko, Sophia Tsong Huey Chew, Azarinah Binti Izaham, Toshiya Koitabashi, Varinee Lekprasert, Suraphong Lorsomradee, Judy Munday, Yasuko Nagasaka, Nicholas Ralph, Raveenthiran Rasiah, Shunsuke Tachibana, Suwimon Tangwiwat, Sadhana Trivedi, Michiaki Yamakage, David Sturgess

**Affiliations:** 1 Medical College of Wisconsin, Froedtert Hospital, Milwaukee, WI USA; 2 Singapore General Hospital, Singapore, Singapore; 3 Faculty of Medicine, University Kebangsaan, Kebangsaan, Malaysia; 4 Tokyo Dental College Ichikawa General Hospital, Ichikawa City, Japan; 5 Department of Anesthesiology, Faculty of Medicine, Ramathibodi Hospital, Bangkok, Thailand; 6 Chiang Mai University, Chang Mai, Thailand; 7 School of Health, University of the Sunshine Coast, Sippy Downs, Queensland, 4556 Australia; 8 Tokyo Women’s Medical University Hospital, Tokyo, Japan; 9 Avisena Specialist Hospital, Shah Alam, Malaysia; 10 Sapporo Medical University, Sapporo, Japan; 11 Department of Anesthesiology, Faculty of Medicine, Siriraj Hospital, Siriraj, Thailand; 12 Solventum, Dublin, Ireland; 13 Princess Alexandra Hospital, The University of Queensland, Brisbane, QLD Australia

**Keywords:** Patient warming, Perioperative normothermia guidelines, Perioperative hypothermia prevention, Continuous temperature monitoring, Inadvertent perioperative hypothermia, Core body temperature monitoring, Normothermia guideline implementation, Single-use warming blanket, Free hosing, Anaesthesia

## Abstract

**Background:**

Inadvertent perioperative hypothermia, a common complication secondary to anaesthesia and surgical exposure, affects patients globally and is associated with adverse outcomes. Interventions to prevent perioperative normothermia include consistent core temperature monitoring and active warming strategies. Forced-air warming is the key active warming strategy recommended in guidelines and requires consistent perioperative application to prevent hypothermia. Implementation of guidelines varies across the Asian Australasian Regional Section (AARS), characterised by diverse healthcare systems and resource constraints. An advisory panel was convened to identify regional challenges and propose recommendations for perioperative normothermia guideline implementation, including forced air warming.

**Methods:**

An expert advisory panel of 15 healthcare professionals, including anaesthesiologists, registered nurses, and perioperative normothermia research experts from Australia, Japan, Korea, Malaysia, Singapore and Thailand, convened during October 2024. Panellists reviewed relevant literature, shared clinical experiences, discussed challenges, and proposed evidence-based recommendations for safe practices. Meeting outcomes are summarised in this publication.

**Discussion:**

Obstacles for guideline implementation of perioperative hypothermia prevention were identified and classified into four areas: 1) economic constraints, 2) practical limitations, 3) educational gaps, and 4) environmental challenges. Limited insurance coverage for forced air warming systems, resource limitations, time pressures and inconsistent technology availability were among the identified barriers. The panel recommended leveraging cost-effectiveness studies and conducting future analyses to support hypothermia prevention, emphasising long-term financial benefits of avoiding complications associated with hypothermia. Panel members advocated for the adoption of established guidelines and simplifying and customising them to align with the unique contexts of individual institutions. They recommended improved access to accurate non-invasive monitoring devices within each institution, and for training regarding continuous temperature monitoring. To enhance knowledge among surgical teams, the panel emphasised the importance of establishing ongoing comprehensive training and structured workflow systems, supplemented by regular audits. The panel identified incorrect use of forced air warming systems as a significant barrier to perioperative hypothermia prevention. Members stressed the necessity of incorporating proper usage training into a comprehensive educational program detailing correct application of forced air warmers, infection control, and liability awareness. This work aims to enhance patient safety, improve clinical outcomes, and reduce economic burdens across diverse AARS healthcare systems.

## Background

Inadvertent perioperative hypothermia, a common complication secondary to anaesthesia and surgical exposure, affects patients globally (Ruetzler and Kurz 2018). Perioperative hypothermia is associated with adverse outcomes, including surgical site infections (Balki et al. 2020; Beilin et al. 1998), coagulopathy (Rajagopalan et al. 2008), delayed wound healing (Scott et al. 2015), and complications requiring increased medical intervention (Balki et al. 2020; Frank et al. 1997; Sheffield et al. 1996; Lenhardt et al. 1997; NICE 2016), which significantly impact patient recovery, cost and quality of life (Sessler 2016; Conway et al. 2019). Evidence-based perioperative normothermia guidelines (NICE 2016; WHO 2018; ASPAN 2024; Fawcett et al. 2020; AORN 2024; ACORN 2023; ANZCA 2025), consensus statements (Munday et al. 2023a), and regional standards (Torossian et al. 2015; Badia et al. 2020) recommend maintaining core body temperature ≥ 36.0 °C (Table 1).

**Table 1:** Summary of perioperative normothermia guideline statements

Organization	Key Recommendations
World Health Organization (WHO 2018)	Forced air warming to reduce SSIs; continuous temperature monitoring to prevent inadvertent perioperative hypothermia
Enhanced Recovery After Surgery 2020 (Fawcett et al. 2020)	Integrate forced air warming in enhanced recovery protocols; maintain core temperature near 36.5 °C intraoperatively
National Institute for Health and Care Excellence (NICE 2016)	Maintain core temperature ≥ 36.0 °C; use forced air warming pre-, intra-, and postoperatively; monitor temperature every 30 min intraoperatively
Australian and New Zealand College of Anaesthetists (ANZCA 2025)	Recommend forced air warming and warmed fluids; monitor core temperature
Association of periOperative Registered Nurses (AORN 2024)	Prewarm patients with active warming for ≥ 10 min; use warmed IV fluids for > 1L; continuous temperature monitoring
American Society of PeriAnesthesia Nurses (ASPAN 2024)	Ensure normothermia (≥ 36.0 °C) throughout perioperative phases; forced air warming as primary warming method
Australian College of Perioperative Nurses (ACORN 2023)	Aligns with NICE; emphasise forced air warming and temperature monitoring in all surgical settings

*IV* intravenous, *SSI* surgical site infection

Interventions to prevent perioperative normothermia include consistent core temperature monitoring and active warming strategies. Forced-air warming is the key active warming strategy recommended in guidelines and requires consistent application prior to and during surgery to prevent hypothermia (Rajagopalan et al. 2008; WHO. 2018; Madrid et al. 2016; Ciftci et al. 2024; Ho and Tan 2011).

**Fig. 1:**
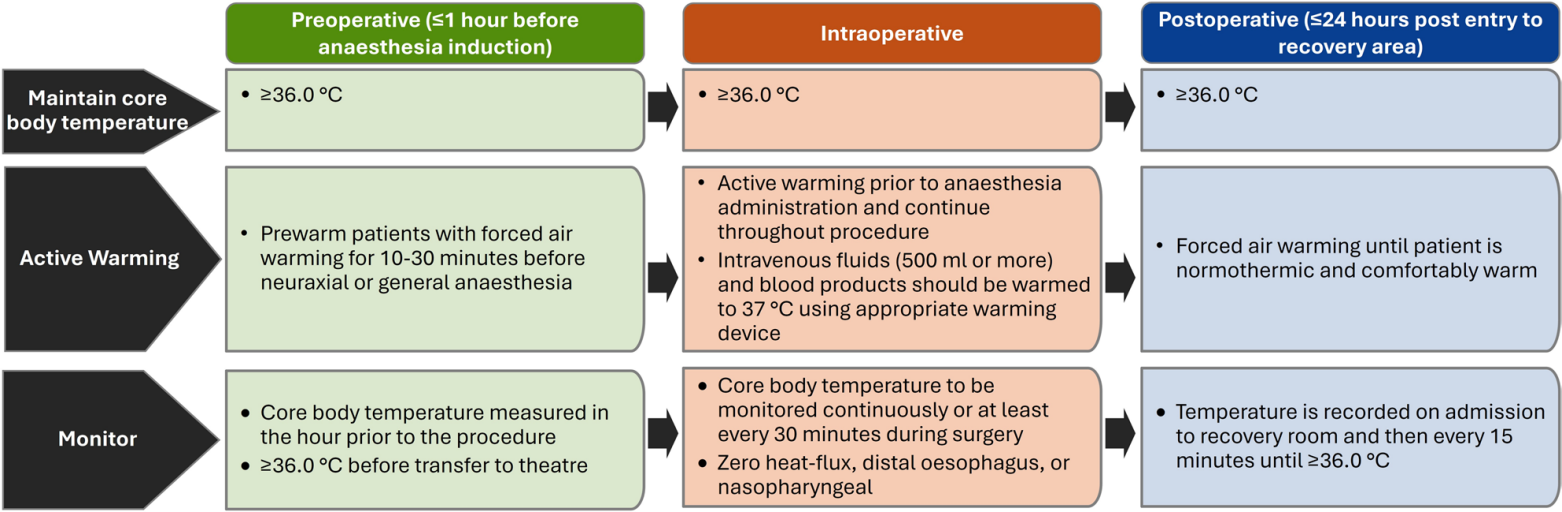
Warming and monitoring recommendations synthesised from published national and international guidelines, and consensus recommendations NICE 2016, Global Guidelines/WHO 2018, ASPAN 2024, Fawcett 2020, AORN 2024, ACORN 2023, Munday 2023, Torossian 2015

Figure 1 presents a flowchart that summarizes instructions for warming and monitoring, synthesised from guidelines and consensus documents.

Implementation of guidelines for hypothermia prevention varies globally. The Asian Australasian Regional Section (AARS), aligned within the World Federation of Societies of Anaesthesiologists and encompassing Asia, Australia, New Zealand, and Western Pacific Islands, faces unique compliance challenges due to its diverse healthcare systems and economic disparities (Yi et al. 2017; Koh et al. 2021).With a population exceeding 4 billion, this region has a varied healthcare infrastructure ranging from advanced systems in Australia and Singapore to resource-limited settings in Southeast Asia (The World Bank 2023). AARS countries exhibit significant variation in perioperative normothermia guideline uptake, which may be driven by economic constraints, but potentially also by differences in awareness and attitudes towards the importance of hypothermia prevention. These differences suggest the need for tailored solutions to improve implementation of forced air warming and temperature monitoring across AARS (Koh et al. 2021).

This article arose out of the growing need to address AARS-specific barriers to perioperative hypothermia prevention. By convening an expert panel to identify regional challenges and propose actionable recommendations for implementation of forced air warming, this work aims to enhance patient safety, improve clinical outcomes, and reduce economic burdens across diverse AARS healthcare systems.

## Methods

The advisory panel convened in Sapporo, Japan (October 2024) and comprised 15 purposively selected healthcare professionals, including anaesthesiologists, registered nurses, and perioperative normothermia research experts from Australia, Japan, Korea, Malaysia, Singapore and Thailand. The panel meeting was sponsored by Solventum. Panel members were selected by Solventum for their expertise and contributions to perioperative temperature management, experience with forced air warming systems, and prior involvement in developing global or local perioperative normothermia standards. The in-person meeting was chaired by two moderators (J.S.K. and D.S.) and was digitally recorded and transcribed in real time to facilitate follow-up.

During the meeting, panellists reviewed relevant literature and shared clinical experiences, discussing challenges and proposing evidence-based recommendations for safe perioperative normothermia practices. A medical writer (K.B.) summarised the discussions into an outline, which was distributed to panel members for feedback. Manuscript refinement occurred through remote online meetings and email exchanges, with multiple rounds of input to revise drafts. The final manuscript draft was approved by all panel members.

## Discussion

The panel discussion identified common and unique obstacles for guideline implementation of perioperative hypothermia prevention that were classified into four key areas: 1) economic constraints, 2) practical limitations, 3) educational gaps and 4) environmental challenges. Challenges as well as recommendations for overcoming them are outlined below.

### Economic constraints

The panel highlighted economic constraints as a central barrier to guideline adoption, driven by limited insurance coverage for forced air warming systems and resource limitations, particularly in smaller hospitals (Koh et al. 2021; Torossian 2007; Waeschle et al. 2015).This issue is evident worldwide, and includes countries such as Singapore, Malaysia, Thailand, Philippines, India and South Korea, as reported by Koh et al. (2021). The approach to funding and reimbursing consumables, such as forced air warming blankets, varies for each AARS country and health service, and can pressure individual practitioners to try and save money by not buying consumables in high volumes. This can lead to a lack of availability of commercial products in the operating theatre and along the patient care pathway.

To address this, the panel recommended using cost-effectiveness evidence to advocate for hypothermia prevention, citing studies showing savings (Conway et al. 2019; Zucconi et al. 2022; Monzani et al. 2020). While costs of warming systems, including forced air warmers, radiant blankets and other devices, increase primary health care expenditures, this may be far outweighed by prevention of adverse outcomes associated with perioperative hypothermia, including SSIs, morbid cardiac events and transfusions. For instance, such complications have been reported to cost Australia AUD $1.3 billion annually (Ralph et al. 2020), and results from a model-based cost-effectiveness analysis of implementation of a thermal care bundle (assessing risk, recording temperature and actively warming) (Duff et al. 2017) showed simultaneous reduction in costs and increased quality-adjusted life years in 88.1% of simulations (Conway et al. 2019).

To better tackle fiscal challenges, particularly among those who control funding, panel members identified the need to conduct additional cost analyses studies. They also proposed engaging policymakers for broader insurance coverage and collaborating with medical societies to emphasise long-term economic benefits.

### Practical limitations

Practical limitations emerged as a critical theme, encompassing time pressures, restricted surgical site access, and inconsistent availability of technology. A fundamental barrier emphasised during discussion was the lack of effective and consistent temperature monitoring, especially during induction and early intraoperative phases. In many settings, particularly in resource-limited hospitals, core temperature monitoring is not performed routinely or accurately (Yi et al. 2015; Yuksek et al. 2020; Duff et al. 2014). Without reliable monitoring, hypothermia may go unrecognised and untreated (Munday et al. 2023b). Panel members highlighted the need for broader access to accurate non-invasive monitoring devices, such as zero-heat-flux sensors, and for strengthening education on the importance of continuous temperature monitoring across all perioperative phases. Support from national societies to incorporate temperature monitoring into routine vital sign assessment and safety checklists was also recommended. Challenges related to practicality are listed in Table 2 with recommendations for proactively addressing these barriers.

**Table 2:** Practical challenges of maintaining perioperative normothermia

Practical Challenges	Recommendations
Low clinical priority (time and efficiency pressures)	• Integration into surgical safety checklist
Difficulty accessing surgical site through blanket	• Increase awareness of various forced air warming blanket configurations that allow access to different areas of the body (Bashaw 2016; Bräuer and Quintel 2009)
Access to warming and monitoring equipment differs across hospitals and countries (Yi et al. 2017; Sento et al. 2017)	• Adapt practice by considering available local resources to achieve desired clinical outcomes • Institutions without pre-anaesthesia areas may consider prewarming on arrival to operating theatre • Consider prioritizing prewarming in institutions without post-anaesthesia care units
Difficulty to use one blanket throughout all care settings: easiest intraoperatively, hardest preoperatively	• Develop system workflow process among all to transition patient with warming blanket throughout all phases of care
Lack of effective, reliable, and consistent core temperature monitoring, particularly during induction and early intraoperative period	• Ensure access to accurate non-invasive core temperature monitors across perioperative settings (e.g., zero-heat-flux) • Promote importance of continuous monitoring and appropriate device selection (Bashaw 2016) • Encourage national societies to prioritise temperature monitoring with minimum monitoring standards
Perception of unsustainability with single use disposables (waste)	• Conduct analysis on efficacy of various warming modalities to validate clinical product selection, i.e., understand heat transfer rates or conduct temperature review • Perform health economics analysis factoring in cost of complications to understand overall resource impact • Partner with country-specific recycling initiatives to reduce landfill impact and promote circular economy models for single-use medical devices (Hoveling et al. 2024) • Reusable probe for noninvasive core temperature monitor needed
Documentation systems may not enable temperature recording which can impact repeated temperature monitoring	• Establish quick, easy way to enter hypothermia interventions and regularly record patient’s temperature in medical record (Bashaw 2016) • Add task to pre-op checklist • Include on post-anaesthesia care unit post-op form • Explore technologies that assist in capturing data

### Educational gaps

Another prominent discussion theme was knowledge gaps among surgical teams regarding warming techniques and their cost-effectiveness, which undermines adherence to best practices (Koh et al. 2021; Burger and Fitzpatrick 2009; Weirich 2008; Shorrab et al. 2007). Strategies suggested by panel included provision of simplified, institution-specific guidelines, comprehensive training, and workflow systems with audits, as outlined in Table 3. Training should emphasise hypothermia risks, use of multimodal approaches (e.g., forced air warming and warmed fluids), and quality-of-life impacts (Munday et al. 2019; Braithwaite et al. 2017).

**Table 3:** Priorities for promoting importance of perioperative hypothermia prevention

**Adopt guidelines and adapt per institution (**Munday et al. 2025**)**	**Promotion of perioperative hypothermia prevention**	**Establish workflow and follow-up**
• Clear, simple temperature management guidelines per institution • Form consensus definition of “perioperative hypothermia” • Identify key gaps and use a phased approach to adoption • Use already established evidence-based guidelines as basis • Contextualise to local resources (Koh et al. 2021) • Align guidelines with stakeholders	• Combine existing programs into comprehensive training • Include planning, updated product training, and safety education (AORN 2024) • Highlight hypothermia risks and multimodal approach (Munday et al. 2019) • Share information on quality-of-life and impact on cost control • Emphasise teamwork and consequences of inaction (AORN 2024)	• Designate stakeholders for implementation • Promote clear communication across perioperative team and with patients (ACORN 2023) • Set monthly goals, provide audit with timely feedback, and conduct follow-up surveys (Wagner 2003)

Several panel members proposed strategies for partnering with industry to help ensure perioperative normothermia. The panel emphasised the pivotal role of medical technology companies in addressing AARS-specific barriers through innovation and collaboration (Houlding et al. 2021). Beyond educational support, industry may drive guideline implementation by developing cost-effective forced air warming systems, sustainable disposables, and standardised data tools, addressing economic and practical challenges. The panel suggested that industry sponsored research may facilitate multi-site studies and engage in guideline discussions to align solutions with AARS needs, fostering interdisciplinary partnerships to enhance patient outcomes.

### Correct use of forced air warming

Panel members identified incorrect use of forced air warming as a barrier to safe perioperative hypothermia prevention (Bräuer and Quintel 2009). This highlighted the need to incorporate proper usage training into a comprehensive educational program that emphasises correct use of forced air warmers, infection control, and liability awareness. Forced air warmer system manufacturer instructions state among other directives that warming blankets are for single patient use, that the warming unit hose should not be used alone, and that the hose should be connected securely to the dedicated warming blanket (3M. 2016). Forced air warming blankets are designed to distribute heat from the warming unit hose and transfer that heat evenly, diffusing warm convective air around the patient’s skin. The efficacy of forced air warmer systems is largely contingent on the design of the blanket, with an optimal blanket characterised by minimal temperature differential across its surface (Bräuer and Quintel 2009; Ji et al. 2024).

Unintended use, such as free hosing and reusing blankets, can lead to serious adverse events including thermal injury and cross-contamination, both of which negatively impact patient outcomes (Wagner 2003; Uzun et al. 2010; Wu 2013; Chung et al. 2012; Mehta 2013; ECRI 1999; Marders 2025). Adhering strictly to manufacturer instructions regarding use of warming units with respective blankets or gowns is critical for avoiding potential harm. Using the warming system as intended (e.g., single-patient blankets, secure hose connections), is essential for system function, minimizing complication risks and achieving guideline-reported reductions in negative surgical outcomes (Bräuer and Quintel 2009; Nemeth et al. 2021).

Panel members discussed possible factors that may contribute to the improper use of forced air warming in AARS, including economic pressures, lack of product training, and a potential mindset of reuse. While no deaths have been reported, there are several reports of thermal burns during use of forced air warming systems while the hose is disconnected from the blanket (Wu 2013; Chung et al. 2012; Truell et al. 2000). The panel noted that these negative outcomes not only affect patients and families but may also expose healthcare professionals to liability; incorrect use of forced air warming blankets, classified as medical devices, may implicate individual practitioners and nursing management (Wagner 2003).

A misguided belief among some practitioners that forced air warming elevates the risk of SSIs due to disruptions in laminar air flow within the operating theatre further complicates adherence, though evidence confirms the safety of forced air warming when used correctly (Ackermann et al. 2018; Sessler et al. 2011; Kellam et al. 2013; Wood et al. 2014). Panel members emphasised that education may assist to dispel misconceptions about warming and infection. They also advocated for clear institutional policies and operational guidelines to promote uptake, enhance patient safety, and build trust with patients as well as infection control and medical safety departments (Wagner 2003). Table 4 details incorrect use, risks, and solutions.

**Table 4:** Examples of incorrect use of forced air warming (FAW): risks and recommendations for correction

Incorrect use example	Risks associated	Potential solutions
Free hosing (when hose of forced air warming unit is placed underneath surgical drape or blanket of patient’s bed without being attached to appropriate FAW blanket)	• Thermal injury (Wagner 2003; Uzun et al. 2010; Wu 2013; Chung et al. 2012; Mehta 2013; ECRI 1999; Marders 2025)	• Disposable blankets designed specifically for use with forced air warming units are essential in dissipating heat delivered by the device Ensure proper attachment of hose to corresponding warming blanket/gown • Ensure warming hose is not directly contacting patient’s skin during warming therapy • Ensure patient does not lie on warming hose • Ensure hose is not placed under blanket to warm bed when patient is not yet on bed (3M. 2016)
Commingling components with other manufacturer products (using one manufacturer’s warming blanket with a warming unit from different manufacturer)	• Thermal injury • Reduced efficacy	Use only blankets manufactured by same maker of warming unit (designed specifically to work with warming unit) (3M. 2016)
Incorrect attachment of hose to blanket	• Thermal injury (ECRI 1999)	Attach hose to blanket according to manufacturer’s instructions before activating forced air warming unit
Reusing FAW blankets	• Cross-contamination • Medical accidents • Decreased warming performance due to damaged blanket, reduced adhesive strength of fixing tape, or loose hose connection	Each warming blanket/gown is for single patient use
Placing sheet between FAW blanket/gown and patient	• Reduced heat transfer to patient by 67.7% (Stark et al. 2024) • Risk of postoperative complications such as SSI due to reduced warming effect	Perforated side of forced air warming blanket should be in direct contact with patient’s skin (3M. 2016)
Connecting torn or damaged warming blanket/gown to warming unit	• Thermal injury	A new forced air warming blanket should be used for each patient (3M. 2016)
Wet blanket	• Inefficient • Skin maceration • Increased risk of cooling	Important to keep blankets dry
Warming without monitoring patient’s skin and temperature	• Thermal injury • Passive hyperthermia (AORN 2003) • Reduced efficacy • Inability to detect and address hypothermic events	• Monitor patient’s body temp and vital signs while warming (Munday et al. 2022) • Periodically check the appearance of the patient’s skin under the blanket • Air temperature should be reduced or unit turned off if vital signs become unstable or if redness of the skin is observed • Avoid maximum temperature setting for patients with compromised circulation or patients who require warming for an extended period (Truell et al. 2000; Siddik-Sayyid 2010; Siddik-Sayyid et al. 2008)
Incorrect warming of IV or irrigation fluid	• Thermal injury	• Solutions should be warmed or cooled to the temperatures appropriate per surgical need • Fluids should be heated in devices intended for that purpose; microwaves and autoclaves should not be usedas warming devices (AORN 2003) • Follow guidelines provided by healthcare organizations and ensure all staff are trained in correct use of warming devices
Application of warming system over ischemic limb(s)	• Thermal injury (Siddik-Sayyid et al. 2008)	• Do not apply heat to ischaemic limbs (3M. 2016)
Poor device maintenance	• Reduced efficacy	• Bio-engineering checks done on regular basis • Forced air warming devices need to be cleaned, maintained and eventually replaced according to manufacturer instructions

*FAW* forced air warming, *IV* intravenous

### Environmental challenges

Most core heat loss (81%) during surgery has been attributed to the core-to-peripheral redistribution of temperature that occurs post-anaesthesia induction (Siddik-Sayyid et al. 2008; Sessler 2000). While forced air warming is the standard of care for preventing heat loss, the panel identified environmental factors, particularly suboptimal operating room ambient temperatures, as added barriers to maintaining normothermia in AARS surgical settings (Sessler 2000). NICE guidelines recommend maintaining operating room temperatures of at least 21 °C during patient exposure to prevent inadvertent perioperative hypothermia (NICE 2016). However, the panel noted that operating theatres are often kept cooler due to practical considerations, primarily to ensure surgeon comfort (Sessler 2000), rather than prioritizing patient safety by maintaining ambient temperature at a warmer level. This creates a challenge in balancing surgical team comfort with patient perioperative normothermia needs (Deiana et al. 2022). The panel recommended that organizations need to establish institution-specific optimal operating room temperatures, involving all stakeholders (e.g., surgeons, nurses, administrators) in thermostat control decisions.

Additionally, passive warming adjuncts, such as warmed intravenous and irrigation fluids and insulation of non-actively warmed body surfaces, may be helpful and are often underutilised (Brauer and Brauer 2017). Cooler operating theatres, combined with cold infusions or beds, heighten the risks of hypothermia, making forced air warming critical for patient comfort and safety.

## Conclusions

This article outlines some key barriers to perioperative normothermia guideline adherence in AARS countries and offers regionally actionable strategies to support widespread and sustainable adoption of perioperative hypothermia prevention. The identified domains—economic constraints, practical limitations, educational gaps, and environmental challenges—provide a framework for understanding regional challenges in consistently implementing perioperative hypothermia prevention protocols. These findings align with the global literature on perioperative hypothermia prevention but highlight AARS-specific nuances driven by diverse healthcare systems and resource disparities (Koh et al. 2021).

The panel members’ emphasis on limited insurance coverage and funding disparities as obstacles to forced air warmer adoption corroborates prior research identifying economic challenges, particularly in resource-constrained settings (Koh et al. 2021; Waeschle et al. 2015). Fostering region-specific cost analyses aligns with successful strategies in Australia (Conway et al. 2019). Policy engagement with insurers and medical societies could also help bridge this gap, though implementation may vary by country.

Panel members also advocated for informal and formal studies of patient-reported outcomes to help persuade stakeholders to prioritise the issue of hypothermia across areas of patient care. Furthermore, they encouraged research to determine temperature thresholds for heating each person and to further refine the parameters for using forced air warming. Advocacy for institutional and policy-level support is needed to promote long-term cost-effective solutions.

Practical and workflow-related obstacles reflect global challenges but are amplified in AARS’s heterogeneous healthcare infrastructure (Munday et al. 2019, 2022; Bashaw and Keister 2019). Panel member calls for surgical safety checklist integration and local guideline adaptation mirror Enhanced Recovery After Surgery protocols, which have improved compliance in high-resource settings (Fawcett et al. 2020). However, the focus on sustainable disposables and reusable probes addresses AARS-specific sustainability concerns, a unique contribution compared to Western-centric studies (Turner et al. 2024).

Recognition of knowledge gaps among surgical teams aligns with global findings that sharing of knowledge and training assist with guideline uptake (Koh et al. 2021; Wu 2013). Programs should extend beyond equipment training, and emphasise the physiological consequences of hypothermia, its impact on patient outcomes, and the importance of documentation and communication among care teams. This proposed multimodal training approach, emphasizing teamwork and quality-of-life impacts (Munday et al. 2019), echoes successful educational interventions in perioperative care.

Industry-supported initiatives, such as seminars and videos, could amplify these efforts, leveraging partnerships. Industry collaboration has the potential to address multiple barriers, from cost-effective forced air warming designs to standardised data tools. This aligns with trends in low-resource settings, where industry partnerships have driven innovation (Velazquez-Berumen and Manimaran 2016). The panel’s call for multi-site studies and guideline discussions could foster AARS-specific solutions, though ethical oversight is needed to avoid commercial bias.

The panel’s focus on incorrect use of forced air warming highlights a critical patient safety issue. Training on correct forced air warming use and clear institutional policies, as recommended, helps ensure product effectiveness and could mitigate liability risks for practitioners, a novel insight for AARS contexts (Wagner 2003).

Various warming strategies—including passive insulation, warmed intravenous and irrigation fluids, ambient temperature control, and active warming devices such as forced air and resistive heating—should be considered based on clinical context and available resources (Ji et al. 2024). While forced air warming is recognised as the key active warming method, panel recommendations encourage a multimodal approach, with emphasis placed on applying the right warming method, in the right setting, at the right time.

In minimally invasive procedures, such as laparoscopic and robotic surgeries, where surgical exposure is relatively limited, a more selective application of active warming strategies may be considered. In such cases, the use of forced-air warming can be tailored based on patient-specific risk factors, procedural duration, and intraoperative temperature trends. In addition, heated and humidified insufflation systems have been proposed as an adjunct to reduce heat loss during pneumoperitoneum. However, their adoption in routine clinical practice varies across institutions, and while they may contribute to reducing heat loss, they may not consistently provide sufficient warming as a standalone strategy in all clinical scenarios, particularly in longer or higher-risk procedures (Galetin and Galetin 2022; Wittenborn et al. 2022). A multimodal approach—incorporating appropriate warming techniques based on the clinical context—remains important to support maintenance of perioperative normothermia.

The challenge of balancing ambient operating room temperatures for maintaining a patient-safe environment and supporting surgical team performance aligns with global literature on environmental barriers (Dunn et al. 2017). The panel’s emphasis on institutional coordination and shared ownership of temperature regulation policies as well as enhanced forced air warmer use in cooler operating theatres may assist in addressing practical constraints (Sessler 2000). Integrating passive warming measures complements the NICE guidelines but requires infrastructure investment and may not be a viable option for many AARS countries (NICE 2016).

These recommendations have important implications for AARS healthcare systems. Clinically, tailored training and policies can improve uptake and application of perioperative normothermia guidelines. Policy-wise, advocacy for insurance coverage and industry partnerships could address economic barriers. For research, multi-site studies are needed to evaluate region-specific interventions. Our focus on AARS diversities addresses a gap in the global literature on hypothermia prevention, offering a model for other regions.

Despite differences, AARS countries share a common interest in adopting effective technologies to prevent perioperative hypothermia (Yi et al. 2017). Forced air warming systems need to be adaptable—ranging from cost-effective modular units for low-resource environments to advanced, integrated systems for high-resource hospitals (NICE 2016; WHO 2018). Collaborative efforts among healthcare providers, policymakers, and regional societies will be essential to overcoming barriers such as cost, training, and sustainability, while ensuring warming practices remain safe, effective, and regionally appropriate (Wu 2013).

While this article addresses a gap in the literature on hypothermia prevention in AARS countries, we acknowledge limitations. Fifteen panellists representing six AARS countries may not fully capture the region’s diversity. However, the expertise and international standing of experts helped facilitate the group’s awareness of transnational issues in warming. Conversely, the keen interest of panel members on the topic, as well as personal experiences and published work, may shape their perspectives on hypothermia prevention. Moreover, sponsorship of this meeting by a manufacturer of FAW systems introduces inherent conflicts of interest and potential bias. This could raise concerns that panel member recommendations may closely align with the company’s commercial goals. There is also a risk that the panel could be perceived as a “rubber stamp,” where participants may tend toward validating pre-existing company decisions rather than to provide independent, critical feedback. To help mitigate potential bias and ensure that recommendations reflect collective expert opinion grounded in real-world practice, the discussions were moderated by clinical leaders who actively encouraged balanced dialogue and representation of diverse clinical perspectives across AARS regions.

Finally, while all panellists contributed to and approved the text in this manuscript, a rigorous process of establishing consensus was not undertaken. A future initiative to develop an expert panel consensus document specifically targeting AARS countries could further advance practices related to maintaining perioperative normothermia in the region.

## Data Availability

Not applicable.

## Abbreviations

AARS: Asian Australasian Regional Section
ACORN: Australian College of Perioperative Nurses
ANZCA: Australian and New Zealand College of Anaesthetists
AORN: Association of periOperative Registered Nurses
ASPAN: American Society of PeriAnesthesia Nurses
NICE: National Institute for Health and Care Excellence
SSI: Surgical site infection
WHO: World Health Organization
